# High-performance broadband, low-cost metal–semiconductor–metal π-SnS/Si photodetector

**DOI:** 10.1039/d6ra04135c

**Published:** 2026-07-18

**Authors:** Mohamed S. Mahdi, Husam S. Al-Arab, Sara S. Mahmood, Khansaa K. Abbas, Karameh A. Muhammad, Nabaa H. Allawi, Khalil I. Inad, Amnah M. Ibraheem

**Affiliations:** a Scientific Research Commission Baghdad Iraq msaleh196730@gmail.com

## Abstract

The UV-IR broadband photodetectors have attracted considerable attention for applications in telecommunications, environmental dust detection, and thermal imaging. Constructing broadband photodetectors based on multi-material heterostructures with multiple active absorber layers presents challenges related to complex interface engineering, increased costs, and reduced reproducibility, thereby limiting their practical applications. Metal–semiconductor–metal (M–S–M) photodetectors employ a single dominant photoactive absorption layer to achieve a broad photoresponse without requiring complex heterojunctions. Furthermore, the M–S–M design may achieve a gain greater than 1, resulting in an external quantum efficiency greater than 100%. This design also has other advantages, including planarity, simplified fabrication, and fast response times. Herein, we report a simple, cost-effective, and high-performance broadband (300–1000 nm) M–S–M photodetector based on a π-SnS film deposited on a silicon substrate *via* chemical bath deposition method. In this design, the π-SnS film serves as the primary photoactive material, while the underlying silicon substrate additionally contributes to photogeneration and carrier transport, particularly in the near-infrared region. The device exhibits a maximum responsivity of 2.42 × 10^3^ mA W^−1^, a detectivity of 5.5 × 10^11^ Jones, high sensitivity (3504), and a fast response time within the (36–90 ms) range. The present photodetector's features, including low-cost, rapid response, and a broad sensitivity range, make it suitable for broadband optoelectronic applications.

## Abstract 2

By combining low cost, rapid response times, and a wide sensitivity range, this photodetector is highly suited for practical broadband optoelectronic applications like telecommunications, dust detection, and thermal imaging. Image created with the assistance of Google Gemini.

## Graphical abstract

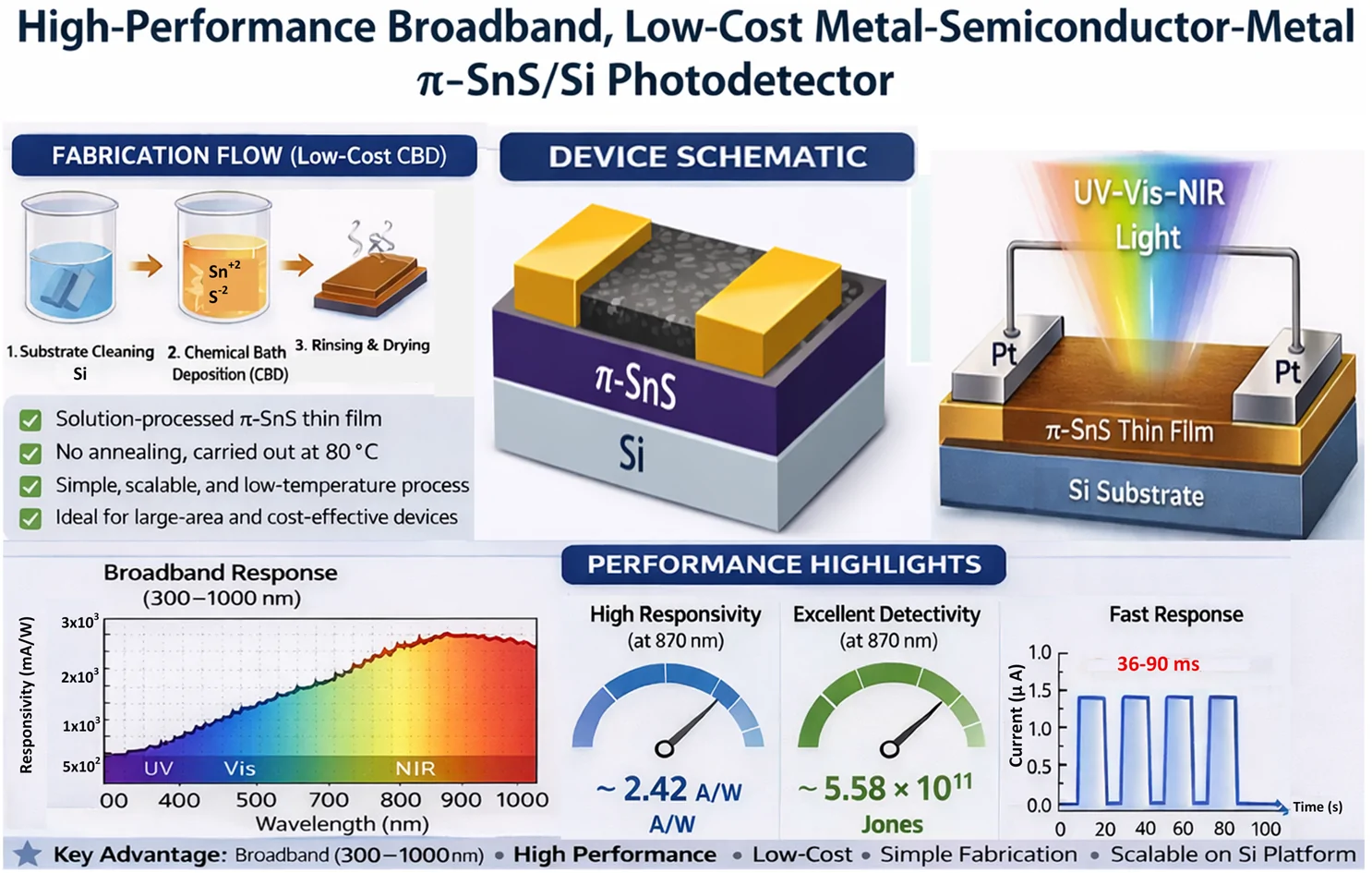

## 1 Introduction

Broadband photodetectors with ultraviolet (UV), visible, and near-infrared (NIR) sensitivities are key elements in modern optoelectronic systems, involving optical communication, environmental monitoring, and imaging technology.^1–3^ It remains a challenge to achieve broadband photoresponse with high responsivity and detectivity using low-cost, scalable process technologies for large-area photodetector applications. Additionally, a heterostructure combining multiple photodetectors of different materials, including In_2_Se_3_/MoS_2_,^4^ BA_2_PbI_4_/GaN,^5^ MoSe_2_/SWCNT,^6^ PtSe_2_/MoS_2_,^7^ PdSe_2_/NbSe_2_,^8^ MoS_2_/graphene/WSe_2_,^9^ MoS_2_/PdSe_2_,^10^ and WS_2_/PdSe_2_ (ref. 11) can broaden spectral sensitivity. However, their preparation is frequently complicated by the interface quality, energy band alignment, and defect-induced carrier recombination. These problems complicate fabrication, increase production costs, and preclude repeatability of the device, especially with multiple active absorber layers or specifically engineered junctions. On the other hand, M–S–M photodetectors with a single dominant absorption-layer configuration are a simple, low-cost route to broadband photodetection. Moreover, M–S–M photodetectors offer distinct advantages, including a simple planar geometry, ease of fabrication, low parasitic capacitance, and a symmetric electrode configuration that minimizes processing complexity, making M–S–M architectures well-suited for low-cost, scalable photodetector integration.^12–14^

Tin monosulfide (SnS) is an attractive IV–VI layered semiconductor for broadband photodetection due to its high optical absorption coefficient, and suitable bandgap (∼1.1–1.75 eV).^15,16^ This is in direct contrast to other emerging materials; for example, chiral perovskites suffer from severe toxicity issues owing to their lead-based components,^17^ while conjugated ladder polymers (cLPs) in organic electronics suffer from poor solubility, synthetic complexity that induces structural defects, and limitations in current characterization techniques.^18^ In addition, SnS is naturally abundant, low-cost, non-toxic, and environmentally stable,^19^ and heavy metals free (*i.e.*, Hg, Cd, and Pb).^20^ SnS films also exhibit pronounced characteristics and are typically considered strongly dependent on the deposition process and experimental techniques.^21–26^ Compared with other emerging two-dimensional materials, SnS combines broadband optical absorption with improved environmental stability and compatibility with low-temperature thin-film deposition processes, making it a promising candidate for cost-effective applications in photodetectors,^27^ solar cells,^28^ thermoelectric generation,^29^ gas sensors,^30^ and humidity sensors.^31^

A considerable amount of literature has been reported on the SnS photodetectors based on SnS/SiO_2_/Si and SnS/Si. In these studies, SnS nanostructured films have been synthesized using relatively costly and complex deposition methods, including chemical vapor,^32,33^ RF sputtering,^34^ atomic layer,^35,36^ thermal evaporation,^37^ pulsed laser,^38^ as well as low-cost and simple methods, including hydrothermal method,^39,40^ microwave,^41^ chemical bath deposition (CBD).^42^ Despite the previous SnS/Si photodetectors being fabricated using simple, low-cost methods, they exhibited relatively low responsivity (<500 mA W^−1^) and detectivity (10^10^ Jones).^34,37,40–42^ Despite using CBD as a simple and cost-effective technique, this earlier research^42^ did not yield promising outcomes: low responsivity (0.388 mA W^−1^) and detectivity (6.48 × 10^6^ Jones). In this study, a broadband M–S–M π-SnS photodetector operating in the 300–1000 nm range was fabricated on a Si substrate using a deposited film *via* a low-cost CBD method. The device exhibits a high responsivity of 2.42 × 10^3^ mA W^−1^ and a detectivity of 5.58 × 10^11^ Jones. Most importantly, the present device does not require a complex multilayer heterojunction configuration, demonstrates the success of a straightforward device architecture, and exceeds the performance of previously reported SnS-based photodetectors.^34,37,40–42^ Additionally, substrate engineering plays a crucial role in the performance of SnS-based photodetectors. This research shows markedly improved results compared to SnS photodetectors on glass substrate (10^−2^ mA W^−1^)^43^ and PET substrate (10^−3^ mA W^−1^),^44^ under identical fabrication and illumination conditions. The results show that CBD-deposited π-SnS thin films could be used to develop scalable, low-cost broadband photodetectors.

## 2 Experimental details

**Fig. 1:**
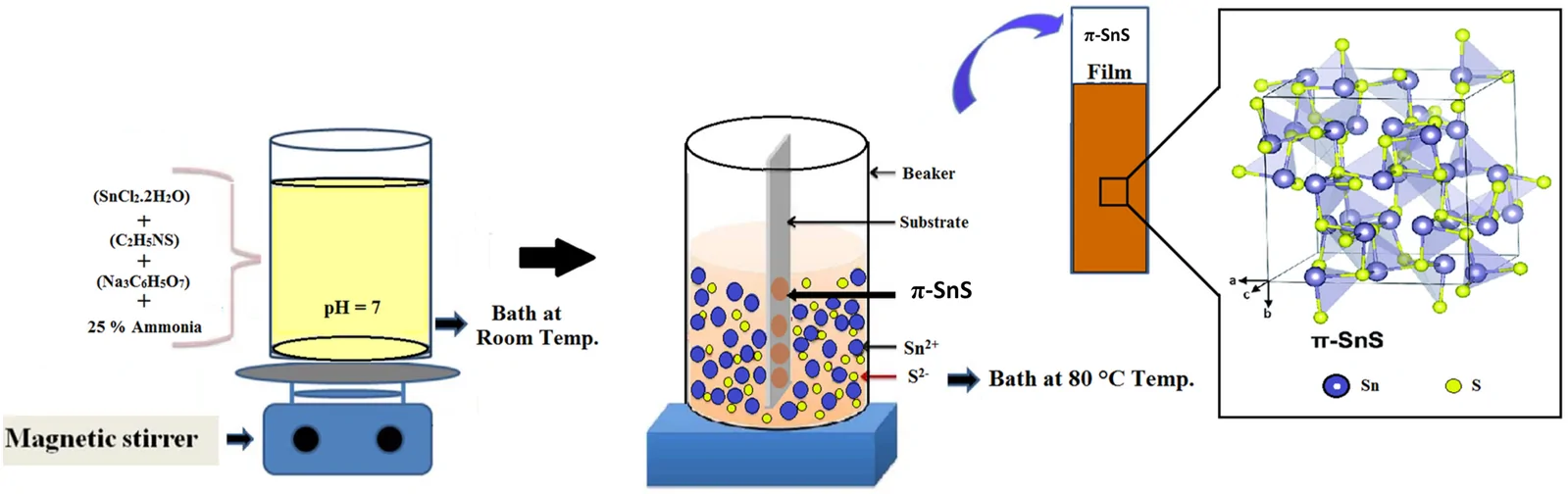
The experimental schematic of the π-SnS film deposition on Si substrate *via* the CBD method.

To deposit the π-SnS film, 0.1 M stannous tin chloride dihydrate (SnCl_2_·2H_2_O) was dissolved in 30 mL of deionized water (DI) by adding 12 drops of concentrated 35% HCl, then ultrasonicated for 30 minutes. Subsequently, 0.2 M dehydrated trisodium citrate (TSC) (Na_3_C_6_H_5_O_7_) and 10 mL of DI water were added to the solution, which was stirred continuously for 90 minutes. Finally, 0.15 M thioacetamide (C_2_H_5_NS) and 10 mL of DI water were added to the solution, which was stirred continuously for 30 minutes. Fig. 1 illustrates the schematic representation of the CBD technique for the deposition of π-SnS thin film onto substrate. The detailed experimental process and characterization techniques for the film are described in the SI.

## 3 Results and discussion

### 3.1 Crystal structure analysis

**Fig. 2:**
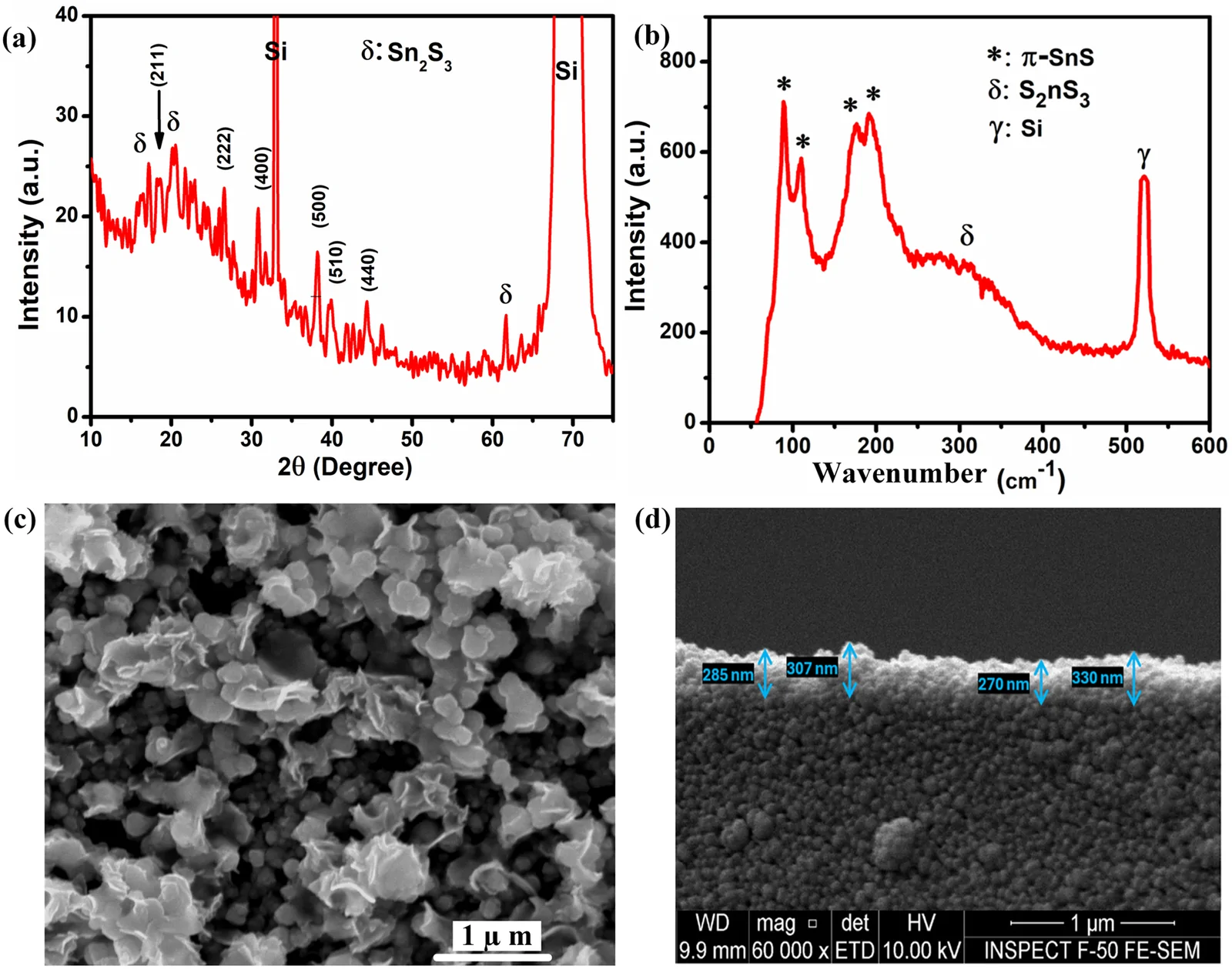
(a and b) XRD pattern and Raman spectrum of the deposited film. (c) The FE-SEM image of the deposited film. (d) Cross-sectional FE-SEM image of the deposited film.

Fig. 2(a) shows the XRD pattern of the π-SnS film grown on a Si (100) substrate. It is clear that relatively low-intensity peaks can be assigned to the (211), (222), (400), (500), (510), and (440) orientations of the cubic π-SnS phase,^45–47^ along with intense Si peaks at 32.98 and 69.13°.^48^ The observed π-SnS peaks align with the JCPDS card PDF 86-9477.^49^ The π-SnS phase fully covers the substrate, as evidenced by the absence of diffraction peaks corresponding to the orthorhombic α-SnS phase. This was confirmed by comparison with the standard reference pattern for orthorhombic α-SnS (JCPDS card no. 00-39-0354), none of whose characteristic peaks were observed in the pattern. In addition, the peaks denoted as (*δ*) were indexed to the Sn_2_S_3_ phase according to JCPDS card no. 01-075-2183,^23^ as a minor phase.

### 3.2 Raman spectroscopy analysis

Raman spectroscopy is effective for analyzing structural and compositional alterations in semiconductors^21^ and is particularly useful for detecting unwanted oxidation phases (such as Sn^4+^ variants). Herein, the main goal is to distinguish SnS from its potential oxidation byproducts, SnS_2_ and Sn_2_S_3_. Fig. 2(b) displays the Raman spectrum of the films in the range of 50–600 cm^−1^ on a silicon substrate. The spectrum indicates that the film exhibits primary vibrational modes at 90, 176, and 192 cm^−1^, consistent with the cubic π-SnS phase.^45–47^ Notably, the characteristic intense peak of pure SnS_2_ (Sn^4+^) at ∼312 cm^−1^ is entirely absent, confirming the suppression of secondary Sn^4+^ phases. The peak at 305 cm^−1^ with low intensity belongs to the Sn_2_S_3_ phase,^50^ and the peak at 111 cm^−1^ belongs to the π-SnS phase.^45^ The strong Raman peak at about 520 cm^−1^ is the vibrational mode of the crystalline Si substrate.

### 3.3 Surface morphology

The FESEM image of the film (Fig. 2(c)) reveals two different morphologies. The initial morphology consists of grains measuring between 59 and 192 nm, while the subsequent morphology is characterized by nanoflakes. The present π-SnS film was deposited under the same pH (7) and bath chemistry (trisodium citrate complexing agent, ammonia-adjusted pH) as our previous study,^51^ in which these conditions were shown to favor a controlled, ion-by-ion (heterogeneous) nucleation pathway over cluster-by-cluster (homogeneous) nucleation, since citrate complexation and gradual ammonia-driven hydrolysis of the sulfur precursor lower the effective bath supersaturation and reduce the heterogeneous nucleation barrier relative to homogeneous nucleation. This ordered, layer-by-layer growth mode allows the film to crystallize directly into the thermodynamically favorable cubic π-SnS phase during deposition, without requiring post-deposition annealing. The observed two-stage morphology is consistent with this growth pathway: the initial grains correspond to early-stage ion-by-ion nucleation, while the subsequent nanoflake formation reflects oriented crystallite growth as deposition proceeds, in agreement with our previous observations under the same bath conditions.^51^ Further details of this growth mechanism are provided in our previous work.^51^

The grain size is critical as it corresponds to the wavelengths of light required for detection. This results in Mie scattering, where the light is scattered within the layer of grains and not reflected externally.^52^Fig. 2(d) illustrates the measured crossed-sectional thickness of the deposited film, which is around 300 nm. This 300 nm thickness is of great importance in a planar M–S–M architecture for the optimization of both optical and electronic performances. It provides an adequate path length optically for effective photon trapping and absorption. The electric field of the depletion region can penetrate the entire active layer in depth distribution effectively due to the thickness of 300 nm electronically. Because the film thickness is kept within the range of the carrier diffusion length, photogenerated carriers have to travel shorter vertical distances to reach the high-field drift regions. This minimizes bulk Shockley–Read–Hall (SRH) recombination pathways. Concurrently, the nanoflakes act as ‘optical antennas’. Their two-dimensional shapes and high aspect ratios significantly increase the surface-to-volume ratio (S/V),^53^ providing more absorption sites, maximizing electron–hole pair generation, and enhancing the overall photoconductive gain of the photodetector.

### 3.4 Photoresponse characteristics

**Fig. 3:**
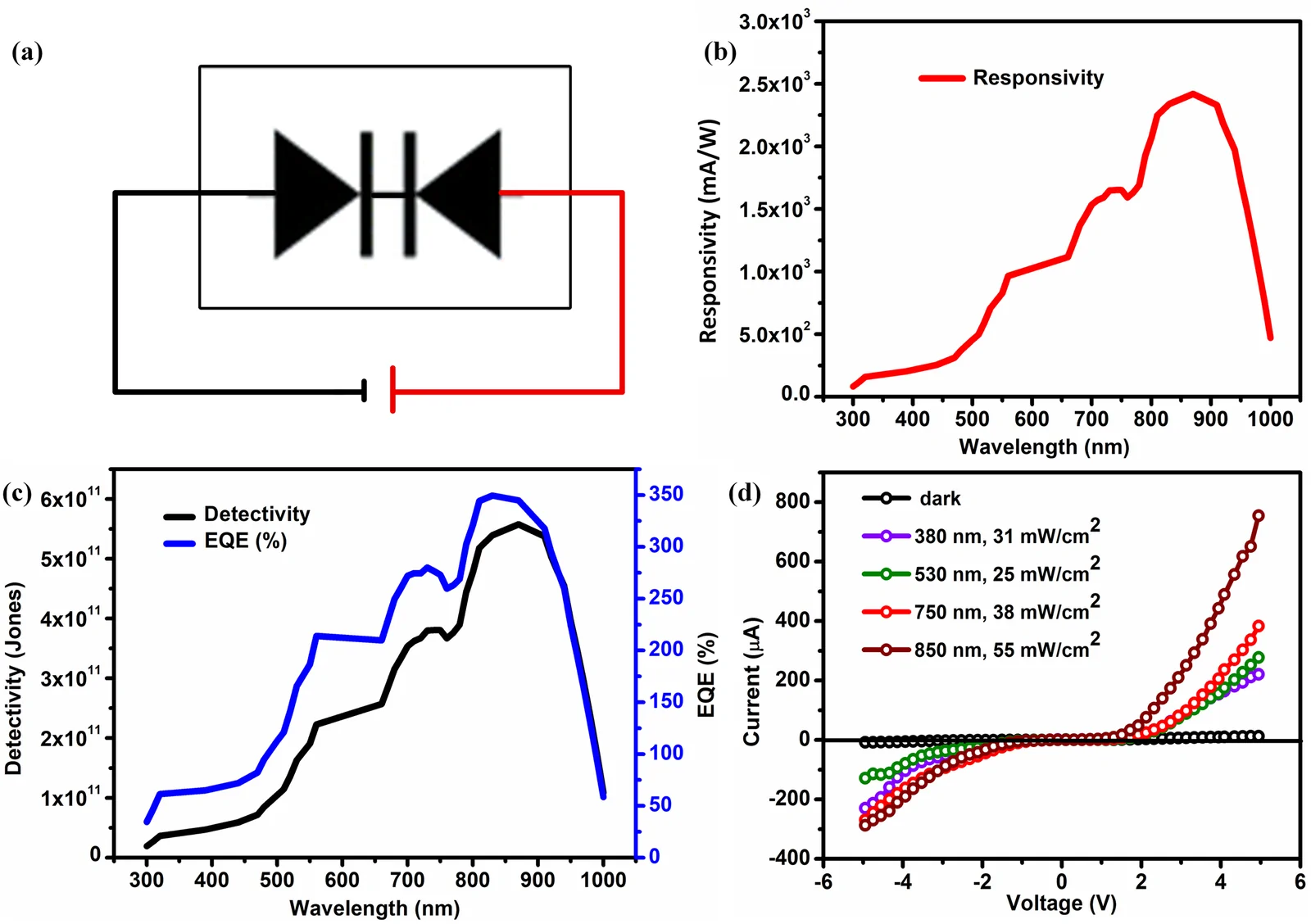
(a) The back-to-back device circuitry. (b and c) The spectral responsivity, detectivity, and external quantum efficiency. (d) The sweeping current–voltage characteristic curves under dark and illuminated conditions at 380, 530, 750, and 850 nm.

As shown in Fig. 3(a), Metal–Semiconductor–Metal (M–S–M) photodetectors have two back-to-back junctions (Schottky or ohmic). The junctions are made by placing pairs of metal electrodes on a semiconductor film. During device operation, one Schottky junction operates under forward bias, and the other under reverse bias. This configuration generates an electric field that separates photogenerated electron–hole pairs throughout the active region. The M–S–M design has many advantages, such as using only one dominant photoactive absorption layer rather than complex heterojunctions. The design is also easy to fabricate, planar, and has a rapid response time due to its low capacitance per unit area.^54^ Additionally, its gain may exceed 1, and consequently, the external quantum efficiency also exceeds 100%.^55^

A wide range of light from ultraviolet to near-infrared was used to study the photoresponse properties of the π-SnS film-based photodetector. Responsivity is an important measure for evaluating photodetector performance and is crucial for determining device sensitivity. The spectral responsivity (*R*_λ_) quantifies how efficiently incident light energy is converted into electrical energy across various wavelengths, expressed in (mA W^−1^). It is defined as the photocurrent (Δ*I*_λ_) produced when light illuminates the effective area (*A*) of the photodetector and is represented as:^56,57^1
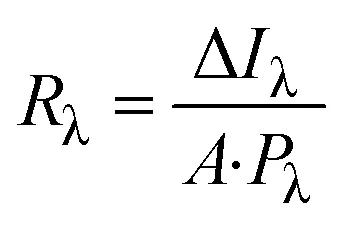
Here Δ*I*_λ_ = *I*_λ_ − *I*_dark_. *I*_λ_, *I*_dark_, and *P*_λ_ present the light current, dark current, and light intensity, respectively.

Fig. S1 shows a schematic of the photoresponse measurement of a broadband π-SnS M–S–M photodetector, and Fig. 3(b) depicts the spectral responsivity of the photodetector across a wavelength range of 300–1000 nm at 5 V bias voltage. The present device demonstrated significant responsivity across the ultraviolet to near-infrared spectrum, with *R*_λ_ values ranging from 3 × 10^2^ to 2.42 × 10^3^ mA W^−1^. Additionally, responsivity increases gradually from the UV region to the visible range and exhibits a pronounced enhancement in the near-infrared region, reaching a maximum of approximately 2.42 × 10^3^ mA W^−1^ at approximately 850–900 nm. The low responsivity in the UV region is attributed to surface recombination losses, whereas the enhanced response in the visible and NIR regions is due to efficient photon absorption and carrier transport. Additionally, the increasing *R*_λ_ value with increasing wavelength may be attributed to a decrease in the energy per photon, meaning you get more photons per watt. Responsivity values greater than 10^3^ mA W^−1^ signify substantial photoconductive gain, possibly resulting from trap-assisted extension of carrier lifetime.^56,58^ A sharp decline in responsivity beyond 900 nm corresponds to the absorption cutoff associated with the active-layer bandgap, as shown in the film's diffuse spectrum (Fig. S2). To evaluate the performance of the fabricated device, Table 1 compares the present photodetector performance with previously reported MSM and P–N junction SnS photodetectors fabricated on Si substrates using various methods.^34,37,40–42^

**Table 1:** The performance of the present photodetector with previously reported M–S–M and P–N junction SnS photodetectors fabricated on Si substrates using various methods^34,37,40–42^

Structure	Method	Illumination conditions	*R* (mA W^−1^)	D* (Jones)	EQE (%)	Ref.
SnS/Si/SiO_2_	RF	808 nm, 5 V	∼75	∼8.5 × 10^9^	∼12	34
M–S–M		0.32 mW, from (Fig. 4(c))				
SnS/Si	Thermal evaporation	532 nm, 5 V	360	3.58 × 10^10^	85	37
P–N junction						
SnS/Si	Hydrothermal and spin coating	150 watts Xe-Arc lamp (1030 nm)	190	9.213 × 10^11^	27	40
M–S–M						
SnS/Si	Microwave	670 nm, −2 V	86.2	7.56 × 10^9^	16	41
P–N junction						
SnS/Si	CBD	Visible radiation, 1 V, 90 mW cm^−2^	3.88 × 10^−1^	6.48 × 10^6^	—	42
P–N junction						
SnS/Si	CBD	5 V, Hg (Xe) lamp. 300–1000 nm	83–2420	1.92 × 10^10^ to 5.58 × 10^11^	34–350	This work
M–S–M						

The most reported devices demonstrate responsivities below 5 × 10^2^ mA W^−1^ and limited spectral coverage. On the other hand, the present M–S–M π-SnS/Si photodetector has a much higher responsivity of about 2.42 × 10^3^ mA W^−1^ and a wide spectral response from 300 to 1000 nm. This enhancement is ascribed to increased light absorption, reduced carrier recombination, and significant photoconductive gain in the SnS film. Notably, the superior performance is achieved without complex heterostructures or expensive fabrication processes, highlighting the effectiveness of the low-cost preparation CDB method employed in this work. Moreover, comparison with our previous SnS film-based photodetectors on glass (10^−2^ mA W^−1^)^43^ and PET (10^−3^ mA W^−1^),^44^ substrates under the same conditions reveals significantly better performance, for the present Si-based device, highlighting the influence of substrate material. This substrate dependence is further supported by our previous systematic comparative study of π-SnS films deposited simultaneously onto PET, glass, and n-type Si substrates,^59^ where the Si-based MSM photodetector consistently exhibited markedly higher responsivity and detectivity than the corresponding PET- and glass-based devices under matched illumination conditions. Furthermore, unlike the linear, ohmic *I*–*V* behavior observed for PET- and glass-based devices, the Si-based device exhibited clearly rectifying, Schottky-like characteristics, indicating the formation of an active SnS/Si heterojunction rather than a purely mechanical interface. This behavior is attributed to the favorable band alignment between p-type π-SnS and n-type Si, which promotes photogenerated carrier separation at the junction, together with partial light penetration through the relatively thin SnS film into the underlying Si, which itself absorbs efficiently across the same near-infrared spectral region due to its narrower indirect band gap. Accordingly, the Si substrate is considered to make a substantive contribution to the photoresponse of the present device, rather than serving solely as a structural support.

External quantum efficiency (EQE) is another figure-of-merit that describes the photoelectric conversion ability of optoelectronic devices and is defined as the number of electron–hole pairs generated by the device per unit time of incident photon illumination. EQE can be expressed as follows:^57,60,61^2
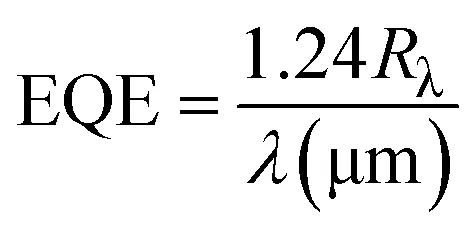
where *λ* is wavelength. Fig. 3(c) displays the EQE in the range of 300–1000 nm. As shown in the figure, the EQE increases gradually in the UV region and rises sharply beyond ∼450 nm, exceeding 100% over a broad wavelength range from approximately 550 to 950 nm. A maximum EQE of ∼350% is achieved at around 870 nm. The presence of a photoconductive gain mechanism is indicated by EQE values exceeding one. The common interpretation of this gain behavior is attributed to the phenomenon known as trap-assisted carrier dynamics. In this model, one type of photogenerated carrier is captured in defect or interface states, whereas the other circulates through the external circuit multiple times before recombination.^62,63^ This process greatly increases the collected photocurrent compared to the incident photon flux. In the present p-type π-SnS film, holes are trapped at interface states associated with the Sn_2_S_3_ secondary phase (confirmed by XRD and Raman spectroscopy), which is known to act as a hole-trapping center in SnS,^43^ an effect reinforced by the polycrystalline nature of the film, whose grain boundaries introduce additional trap states. With holes immobilized, photogenerated electrons—the minority carriers in this p-type material—recirculate multiple times through the external circuit before recombining, producing the observed gain. The discussion of photocurrent–intensity measurements will include direct experimental evidence for this mechanism, such as the wavelength-independent photoresponse time and the sublinear photocurrent–intensity dependence.

Specific detectivity (D*) is a measure of how well a photodetector can detect weak optical signals in the noise presence. It is defined as the normalized light intensity needed to generate an electric signal from the photodetector, equivalent to the photodetector's noise level. D* can be expressed as follows:^56,60,61^3
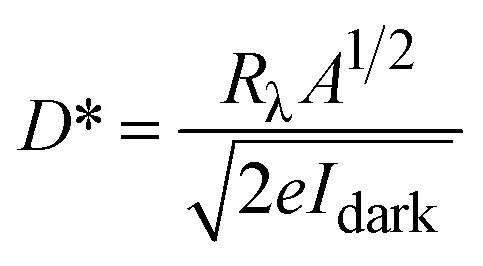
where *e* is the elementary charge. Fig. 3(c) illustrates the D* within the wavelength range of 300–1000 nm. The detectivity spectrum closely aligns with the *R* curve, thereby affirming their strong correlation. Furthermore, detectivity gradually increases with wavelength, reaching a maximum of approximately 5.5 × 10^11^ Jones at 850–900 nm. The improvement in detectivity emerges from the enhanced responsivity linked to the increased EQE, as detectivity is directly proportional to responsivity. The simultaneous peaks in EQE and D* indicate that the enhanced photoresponse is primarily influenced by effective charge transport and gain effects. Both EQE and D* decline markedly beyond approximately 950 nm, which denotes the limit of the device's spectral range. Comparison of EQE and D* of the present photodetector with previously reported SnS photodetectors fabricated on Si substrates,^34,37,40–42^ is shown in Table 1.

The enhancement in *R* and D* for our M–S–M device relative to the previously reported P–N SnS/Si photodiode made from grown film by CBD^42^ is attributed to three compounding factors. First, our MSM structure with back-to-back Schottky contacts exhibits a dark current of ∼10 µA at 5 V (Fig. 3(d)), approximately two orders of magnitude lower than the ∼1 mA dark current reported for the P–N photodiode^42^ (Fig. 5(b)). Since D* is inversely proportional to dark current, this reduction directly contributes to the enhanced detectivity, independent of any difference in responsivity. Second, in our M–S–M device, the exposed Si substrate between the contacts also contributes to photocurrent generation, unlike the single-junction P–N design,^42^ in which photogeneration is confined to the junction depletion region. Third, the nanoflake surface morphology of our SnS film offers a higher effective surface area for light absorption compared to the film's pyramidal microstructural morphology reported previously,^42^ further increasing photogenerated carrier collection.

**Fig. 4:**
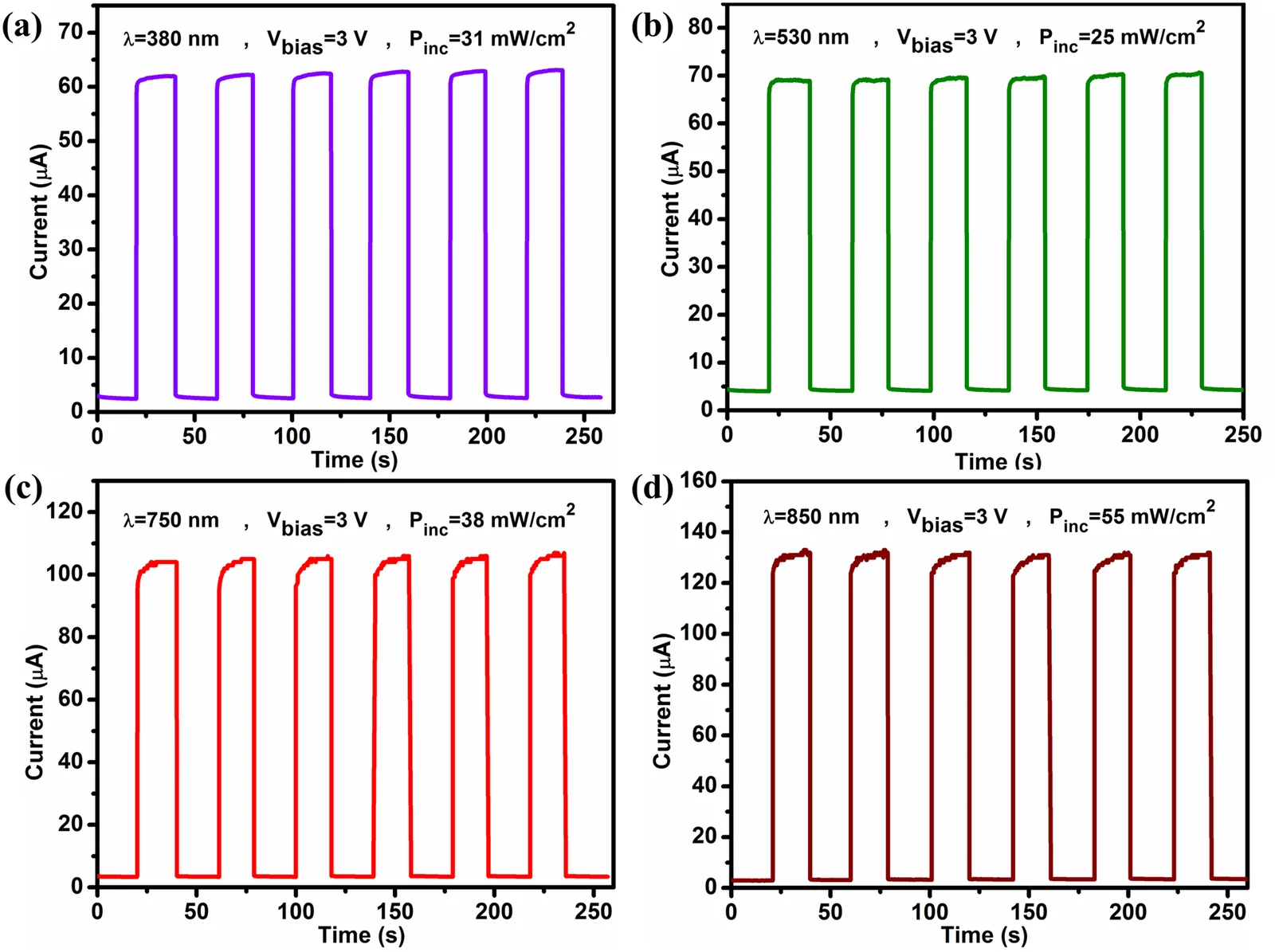
(a–d) The time dependence of the current measured upon 380, 530, 750 nm, and 850 nm illumination and at 3 V bias voltage.

**Fig. 5:**
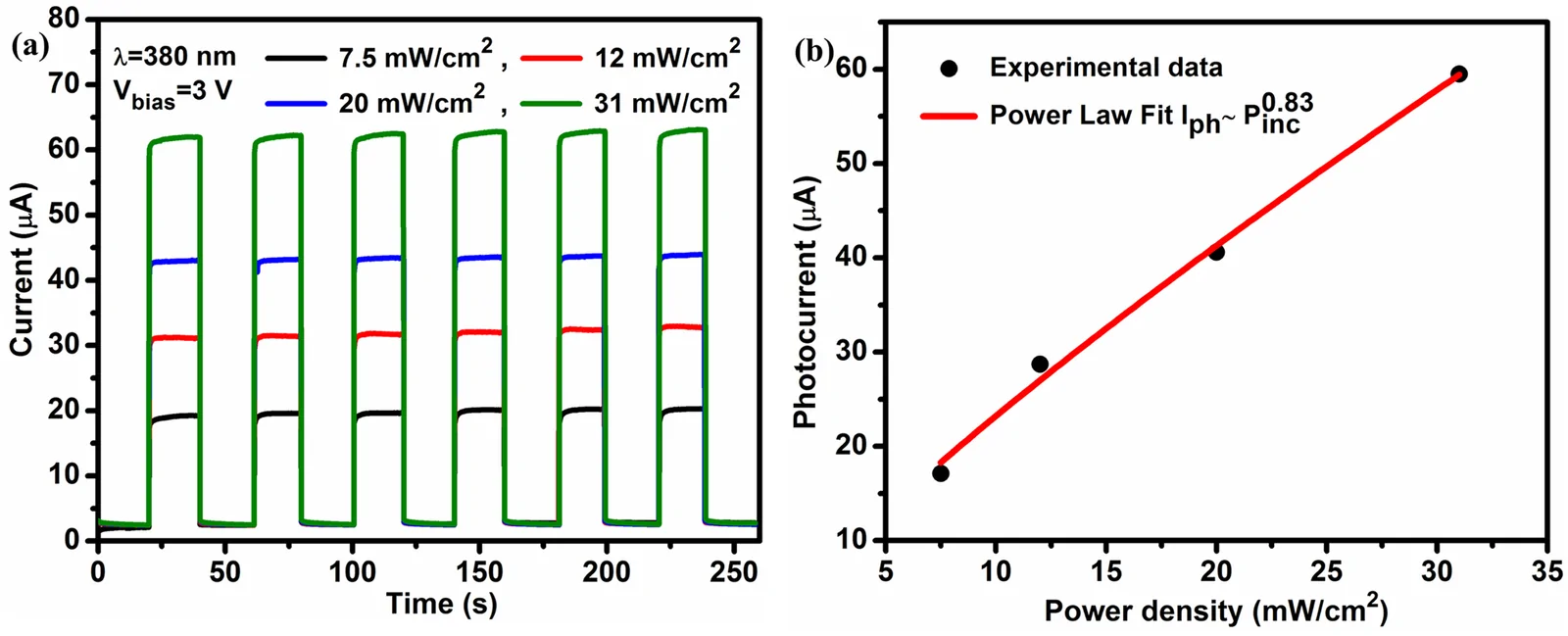
(a and b) The current–time characteristics upon 380 nm illumination and bias voltage of 3 V *versus* light intensity. (b) Experimental and power fitted curves of the photocurrent-light intensity.

Fig. 3(d) shows the device current–voltage (*I*–*V*) characteristics in the dark state and upon illumination through the UV-Vis-NIR range (380–850 nm) using LEDs. The photodetector current increases significantly when exposed to light across all wavelengths relative to the dark current, indicating a pronounced response throughout the UV-Vis-NIR range.

Photoresponse speed and stability are essential for assessing the photodetector performance in technical applications. The photodetector's photoresponse stability was examined upon illumination with four standard wavelengths (380, 530, 750, and 850 nm) across the UV-Vis-NIR range at a 3 V bias, using a 20 s ON/OFF switching cycle, as shown in Fig. 4(a–d). The photoresponse results show that the maximum current remains unchanged across many cycles at all wavelengths, indicating high reproducibility and stability. The current also rapidly reaches a stable level when exposed to light and returns to its original value when the light is turned off, indicating high stability and fast photoresponse times.

To further verify that the reported performance enhancement is not simply a consequence of the differing illumination intensities used for *R* and D* calculation under illumination wavelength 870 nm (0.26 mW cm^−2^ in this work) *versus* visible light illumination (90 mW cm^−2^),^42^ we compare our device's response at 750 nm under 38 mW cm^−2^ with the values reported therein. Under these more comparable conditions, our device exhibits *R* of 15.8 mA W^−1^ and D* of 5 × 10^9^ Jones, *versus R* of 0.388 mA W^−1^ and D* of 6.48 × 10^6^ Jones,^42^ corresponding to an approximately 40-fold higher responsivity and 771-fold higher detectivity even at comparable light intensity. This confirms that the enhancement reflects genuine device-level advantages rather than a measurement-condition artifact.

In practical applications, response speed is an essential parameter. It shows how quickly the device's current changes in response to light. Response time is a measurable physical parameter that quantifies the speed of a device's response, including its rise (*τ*_r_) and fall (*τ*_d_) times. The temporal photoresponse of the device was analyzed by fitting the current–time curve using a single-exponential model expressed as:^39,61^

4 *I* = *I*_d_ − *I*_0_ × exp[−(*t* − *t*_0_)/*τ*_r_]

5 *I* = *I*_d_ + *I*_0_ × exp[−(*t* − *t*_0_)/*τ*_d_]

As shown in Fig. S3, the fitted curve agrees well with the experimental data, yielding an adjusted *R*^2^ of 0.998. The fitted rise times are 65, 76, 91, and 36 ms for 380, 530, 750, and 850 nm, respectively, whereas the fitted fall times are 47, 46, 34, and 34 ms for 380, 530, 750, and 850 nm, respectively. These values are much lower than those of the fabricated photodetector based on deposited SnS film by CBD in a previous study (610 and 580 ms).^42^

Sensitivity (*S*) is a metric utilized to measure the proportional increase in current due to photodetector illumination. As a result, it is an important metric that assesses the performance of the photodetector and is expressed as:^64–66^6
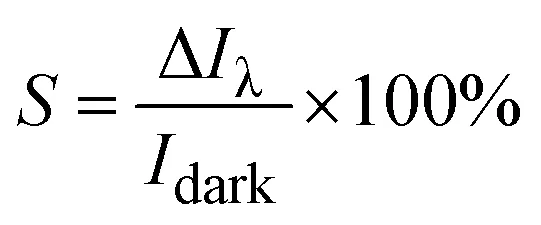


From Fig. 4(a–d), the photodetector's sensitivities were calculated to be 3504, 2887, 1613, and 2444 at wavelengths of 850, 750, 530, and 380 nm, respectively. According to the findings in Fig. 4, the present photodetector is stable and exhibits high sensitivity and fast response across the broadband range.

Additional photoresponse measurements were conducted to assess the photodetector's performance, involving current–time measurements under illumination from a 380 nm LED at varying light intensities with a 3 V bias, as illustrated in Fig. 5(a). It can be noted that increased light intensity increases photon flux density, thereby generating more electron–hole pairs.^15^ The correlation between photocurrent and light intensity, as depicted in Fig. 5(b), can be fit using a power law:^67,68^

7 *I*_ph_ = *CP*^*γ*^

*C* represents the constant of proportionality, and *γ* is a parameter that determines how the photocurrent responds to light intensity.^69^ Fitting eqn (7) to the curve in Fig. 5(b) yields *γ* = 0.83. The comparatively high value of *γ* indicates good equality of the deposited π-SnS film, resulting in excellent photoresponse characteristics. The sublinear exponent could be ascribed to complex processes in semiconductors, including the electron–hole pairs generation, recombination, and trapping.^70,71^

The data presented here directly support the proposed trap-assisted gain mechanism discussed in the EQE. Current–time measurements at 380, 530, 750, and 850 nm (Fig. 4) show a consistent response time of ∼35 ms across the spectral range, which is significantly slower than expected for direct band-to-band transport. This suggests a common trap-mediated origin rather than a wavelength-specific effect. Furthermore, photocurrent–intensity measurements at 380 nm (Fig. 5(b)) show a sublinear power-law dependence (*I*_ph_ ∝ *P*^*γ*^, *γ* = 0.83), consistent with progressive trap filling as incident intensity increases. To exclude measurement artifacts, the illumination spot was matched to the device active area, incident power was calibrated at the sample plane, and the photoresponse was reproducible across repeated ON/OFF cycles with no drift.

The present photoresponse results indicate that the π-SnS film deposited by CBD is promising for fabricating a high-performance broadband M–S–M photodetector.

## 4 Conclusion

In this research, we successfully fabricated a simple and cost-effective high-performance broadband photodetector within the range (300–1000 nm). The photodetector is based on the MSM architecture and a π-SnS film prepared by the CBD method. It demonstrated responsivity 2.42 × 10^3^ mA W^−1^, detectivity 5.5 × 10^11^ Jones, and EQE 350%. It also showed high sensitivity upon illumination of 380, 530, 750, and 850 nm, respectively, with a fast response time of 36 ms. This research provides a simple, low-cost synthesis approach for high-performance broadband photodetectors and reveals that π-SnS is a promising candidate material for next-generation photoelectronic devices.

## Author contributions

Mohamed S. Mahdi: writing – review & editing, writing – original draft, visualization, validation, supervision, resources, methodology, investigation, formal analysis, data curation, conceptualization. Husam S. Al-Arab: writing – review & editing, writing – original draft, resources, formal analysis. Sara S. Mahmood: writing – review, resources. Khansaa K. Abbas: writing – review. Karameh A. Muhammad: writing – review. Nabaa H. Allawi: writing – review. Khalil I. Inad: writing – review. Amnah M. Ibraheem: writing – review.

## Conflicts of interest

The authors declare that they have no conflicts of interest.

## Supplementary Material

RA-016-D6RA04135C-s001

## Data Availability

The data supporting this article have been included as part of the supplementary information (SI). Supplementary information is available. See DOI: https://doi.org/10.1039/d6ra04135c.
