# Supplementary material for: High-performance broadband, low-cost metal–semiconductor–metal π-SnS/Si photodetector
Source: RSC Adv. 2026 Jul 18;16(38):41541–51. doi: 10.1039/d6ra04135c (PMC13379298; doi:10.1039/d6ra04135c)
Supplement: Supplementary file 1 [file RA-016-D6RA04135C-s001.pdf]

Supplementary Information (SI) for RSC Advances.
This journal is © The Royal Society of Chemistry 2026

Supplementary Information

Experimental details

- Film deposition

The n-type single-sided polished Si substrate (crystalline (100), 1–10 Ω·cm) was cleaned using the Radio Corporation of America (RCA) procedure. The π-SnS film was grown on the polished side by the (CBD) technique. To deposit the film, 0.1 M stannous tin chloride dihydrate ($SnCl_2 \cdot 2H_2O$) was dissolved in 30 ml of deionized water (DI) by adding 12 drops of concentrated 35% HCl in an ultrasonic bath for 30 minutes. Subsequently, 0.2 M of complexing agent, dehydrated trisodium citrate (TSC) ($Na_3C_6H_5O_7$), and 10 ml of (DI) were added to the solution under continuous stirring for 90 minutes. Finally, 0.15 M of thioacetamide (C_2H_5NS) and 10 ml of (DI) were added to the solution under continuous stirring for 30 minutes. The solution pH was adjusted to 7 by adding drops of a 25% aqueous ammonia solution. The deposition process was performed at 80 °C for 4 h. Afterwards, the substrate was removed from the beaker, washed with deionized water, and dried naturally. The M-S-M photodetector structure (Pt/π-SnS/Pt was fabricated by depositing the platinum layer (~100 nm) via DC sputtering through an integrated finger mask. The estimated device effective area was 0.17 cm^2.

- Film and photodetector characterization

The crystal structure of the as-deposited film was examined by X-ray diffraction (XRD) using Cu $K\alpha_1$ radiation within a 2θ range of 10 to 70 degrees. The film morphology and cross-sectional thickness were studied using field-emission scanning electron microscopy (FESEM) with the FEI Nova NanoSEM 450 and the Inspect f50 FESEM, respectively. The diffuse reflectance spectrum was recorded using the Cary 5000 spectrophotometer between 300 and 1500 nm. The photodetector's spectral responsivity was determined using a Hg (Xe) lamp as the light source and a monochromator connected to a computer running Cornerstone Utility Programming V4.01 for wavelength selection (Table S1). The photoresponse measurements, which included current-voltage and current-time, were obtained by illuminating light-emitting diodes (LEDs) with peak wavelengths of 380, 530, 750, and 850 nm and using a Keithley 2400 source meter. Fig. S1 presents a schematic of the photoresponse measurement of a broadband π-SnS M-S-M photodetector.

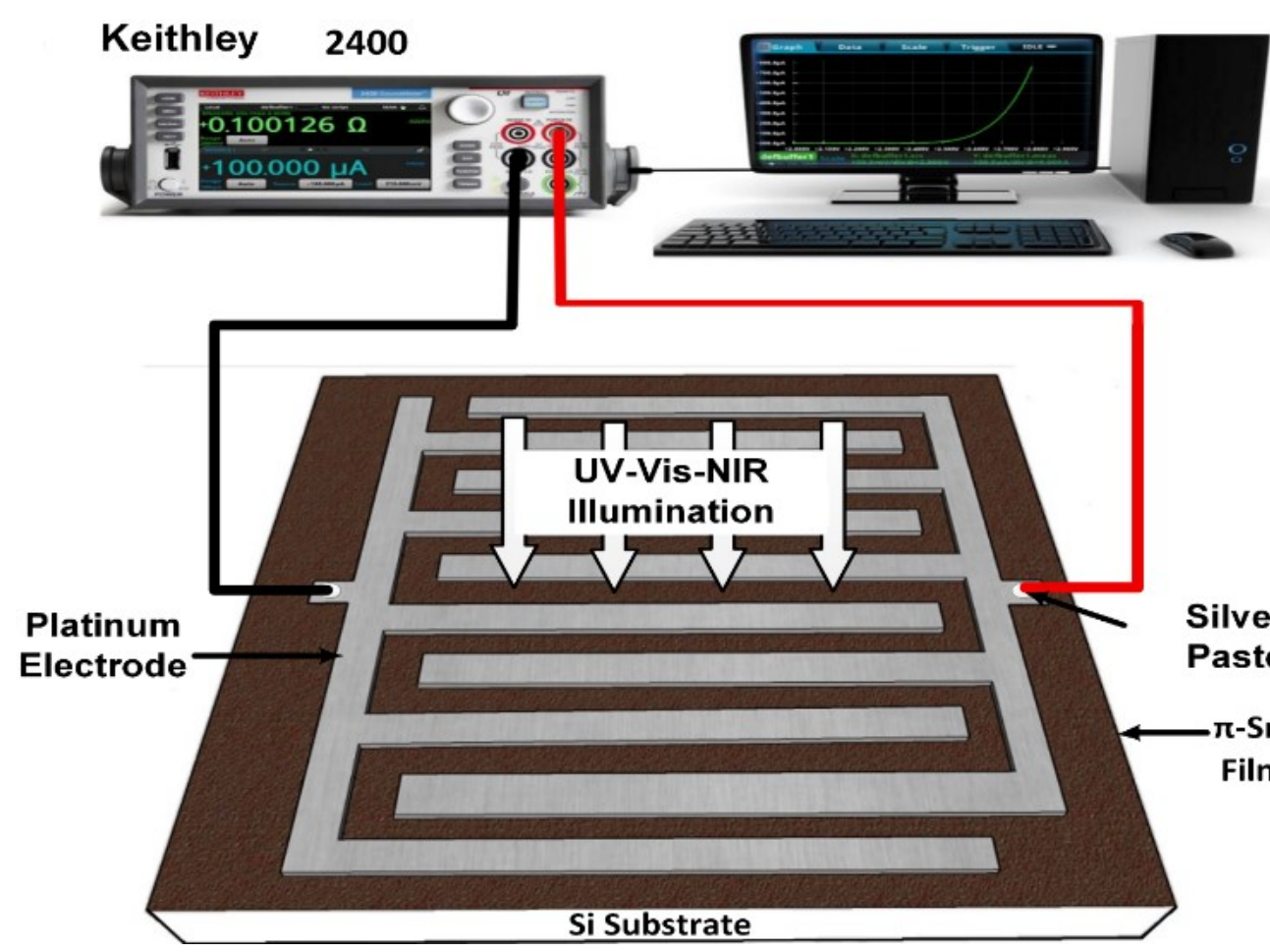

Fig. S1. A schematic of the photoresponse measurement of a broadband π-SnS M-S-M photodetector.

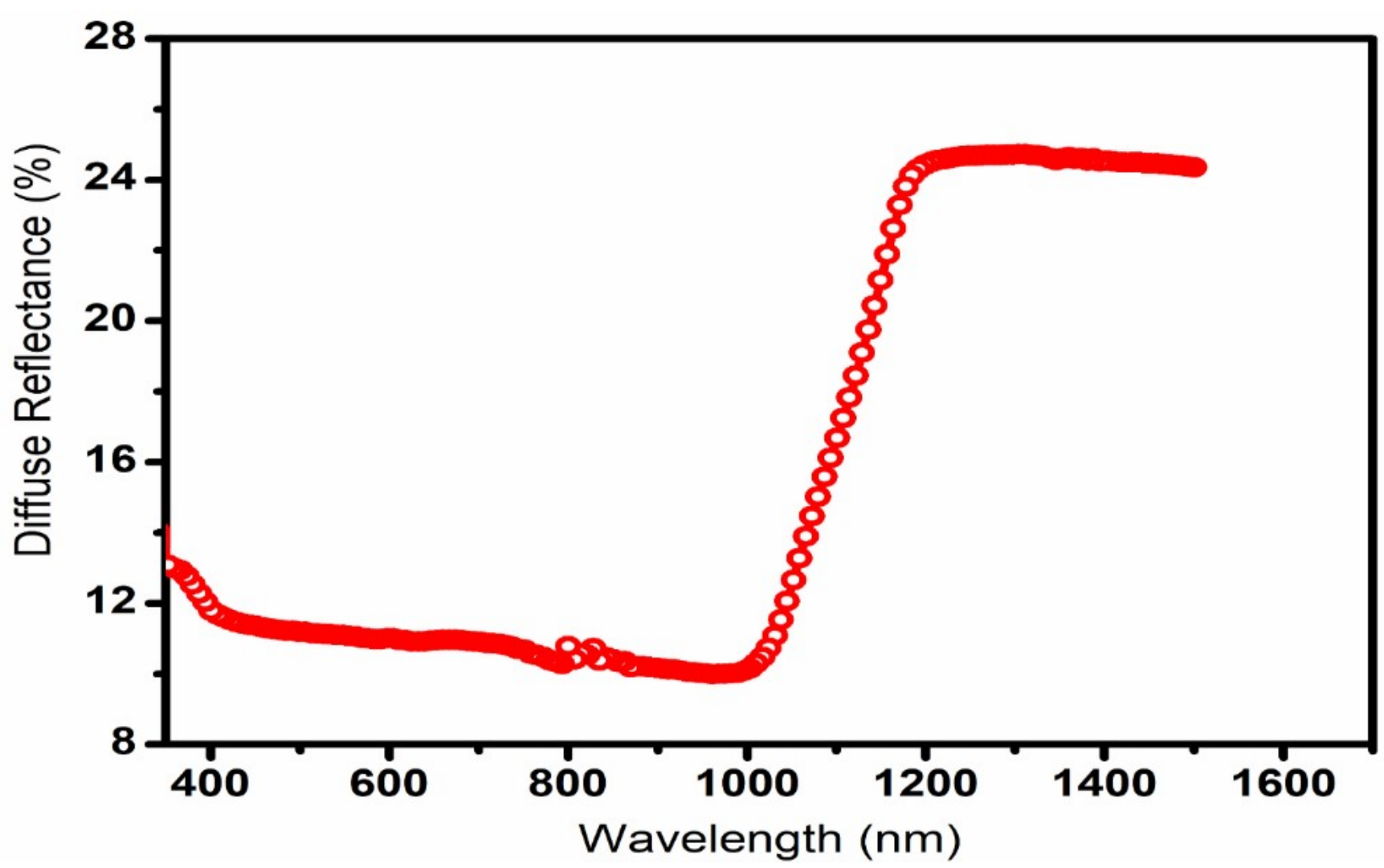

Fig. S2. The diffuse spectrum of the deposited π-SnS film.

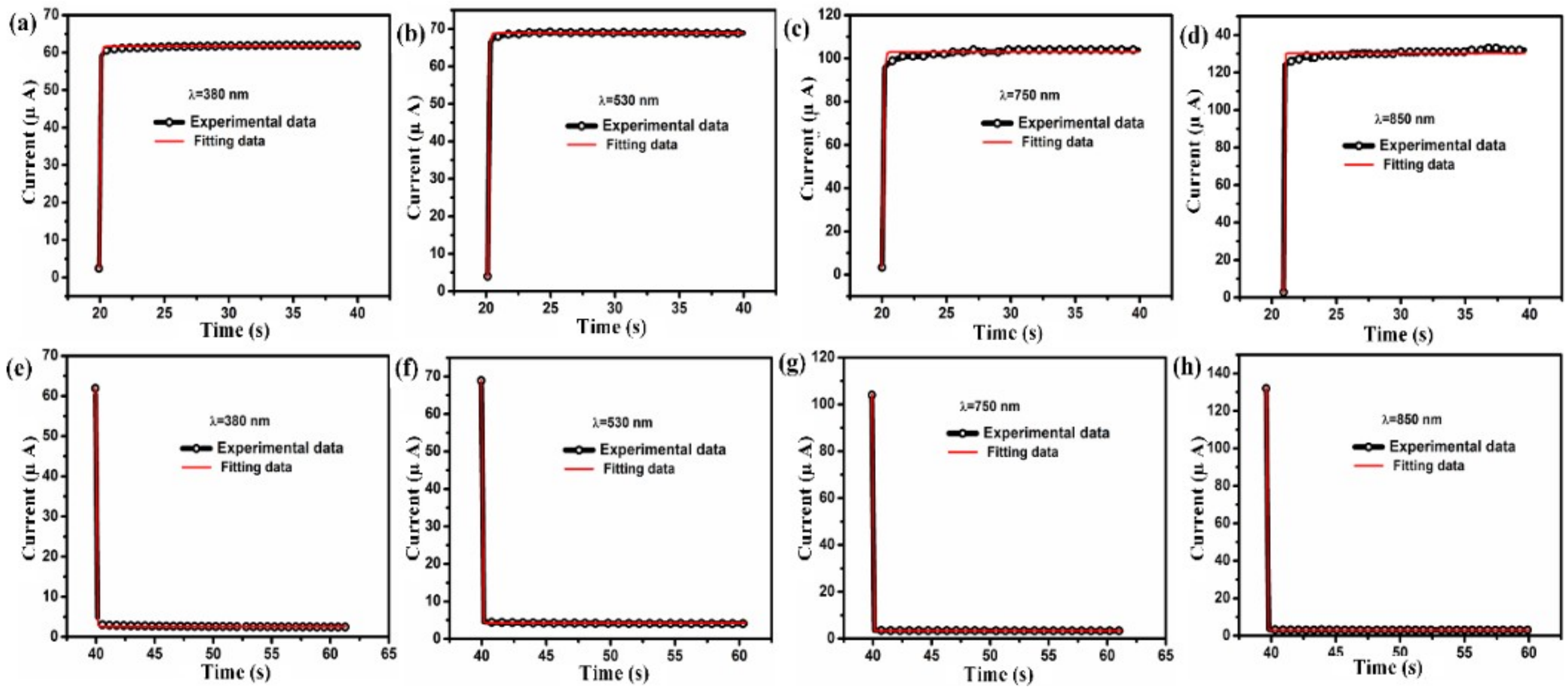

Fig. S3. The fitted current-Time of a single ON/OFF pulse with the experimental data of a broadband π-SnS M-S-M photodetector.

Table S1. Measured Hg (Xe) lamp intensity as a function of wavelength (300–1000 nm).

Wavelength (nm)	Light Intensity (mW/cm^2)	Wavelength (nm)	Light Intensity (mW/cm^2)	Wavelength (nm)	Light Intensity (mW/cm^2)	Wavelength (nm)	Light Intensity (mW/cm^2)
3.00E+02	0.45	5.00E+02	0.17	7.00E+02	0.13	9.00E+02	0.27
3.10E+02	0.58	5.10E+02	0.22	7.10E+02	0.13	9.10E+02	0.35
3.20E+02	0.23	5.20E+02	0.16	7.20E+02	0.1	9.20E+02	0.21
3.30E+02	0.17	5.30E+02	0.27	7.30E+02	0.11	9.30E+02	0.22
3.40E+02	0.13	5.40E+02	1.46	7.40E+02	0.11	9.40E+02	0.35
3.50E+02	0.26	5.50E+02	0.9	7.50E+02	0.12	9.50E+02	0.2
3.60E+02	1.2	5.60E+02	0.27	7.60E+02	0.11	9.60E+02	0.09
3.70E+02	0.61	5.70E+02	1.01	7.70E+02	0.11	9.70E+02	0.25
3.80E+02	0.27	5.80E+02	0.75	7.80E+02	0.09	9.80E+02	0.36
3.90E+02	0.18	5.90E+02	0.42	7.90E+02	0.15	9.90E+02	0.36
4.00E+02	0.6	6.00E+02	0.17	8.00E+02	0.13	1.00E+03	0.21
4.10E+02	0.29	6.10E+02	0.19	8.10E+02	0.12		
4.20E+02	0.21	6.20E+02	0.23	8.20E+02	0.29		
4.30E+02	1.29	6.30E+02	0.27	8.30E+02	0.21		
4.40E+02	0.57	6.40E+02	0.14	8.40E+02	0.11		
4.50E+02	0.23	6.50E+02	0.13	8.50E+02	0.05		
4.60E+02	0.27	6.60E+02	0.14	8.60E+02	0.06		
4.70E+02	0.19	6.70E+02	0.18	8.70E+02	0.26		
4.80E+02	0.19	6.80E+02	0.14	8.80E+02	0.67		
4.90E+02	0.2	6.90E+02	0.14	8.90E+02	0.33		

Calculation for the I–V/LED 850 nm measurement:

Using $R_\lambda = \frac{\Delta I_\lambda}{A.P_\lambda}$

with ΔI_λ = 750 µA (photocurrent at 5 V bias under 850 nm LED illumination, Fig. 3(d)), A = 0.17 cm^2 (effective device area), and P_λ = 55 mW/cm^2:

$R = 750 \times 10^{-6}$ A / (0.17 $cm^2 \times 55 \times 10^{-3}$ W/cm^2) ≈ 0.0802 A/W ≈ 80 mA/W

Calculation for the Hg (Xe) lamp/spectral responsivity measurement:

Using the same relation as the reported with ΔI_λ = 20.1 µA (photocurrent at 5 V bias under Hg (Xe) lamp illumination, Fig. 3(d)), A = 0.17 cm^2 (effective device area), and P_λ = 0.05 mW/cm^2:

$R = 20.1 \times 10^{-6}$ A / (0.17 $cm^2 \times 0.05 \times 10^{-3}$ W/cm^2) ≈ 2.365 A/W ≈ 2365 mA/W

Since responsivity in SnS-based photodetectors scales sub-linearly with incident light intensity (a consequence of trap-mediated photoconductive gain, whereby responsivity increases as intensity decreases), these two measurements yield different R values at the same nominal wavelength and are not directly comparable without accounting for this intensity difference.
